# A cytodiagnosis of adenoid cystic carcinoma of the tracheobronchial tree through a systematic clinical case comparison and analysis

**DOI:** 10.1186/s12890-023-02628-9

**Published:** 2023-09-08

**Authors:** Bing Zhou, Ting Duan, Xianwei Liu, Lizi Peng

**Affiliations:** 1 Department of Pathology, The Second Affiliated Hospital of Jiujiang University, Jiujiang, Jiangxi 332005 China; 2 Department of Pathology, Zhejiang Provincial People’s Hospital, Hangzhou, Zhejiang 314408 China; 3 Department of General Surgery, Jiujiang No.1 People’s Hospital, Jiujiang, Jiangxi 332000 China; 4 Department of Pathology, Jiujiang No.1 People’s Hospital, 48#, Taling Road, Jiujiang, Jiangxi 332000 China

**Keywords:** Adenoid cystic carcinoma, Cytodiagnosis, Tracheobronchial tree, Bronchial brushing

## Abstract

**Background:**

Primary adenoid cystic carcinoma (AdCC) of the tracheobronchial tree is very rare with a high risk for recurrence and metastasis. The diagnosis of AdCC by histologic and immunohistochemical means has been well studied clinically. However, the identification of AdCC by cytologic features remains elusive due to the atypical features the cancer presents. This study aimed to describe the cytologic features of AdCC by using bronchial brushing, which could aid in distinguishing AdCC from other pulmonary carcinomas.

**Methods:**

The cytopathological features of bronchial brushing smears collected from seven cases were histologically diagnosed as AdCC. The defined cytologic features, which could potentially be diagnostic, were systemically analyzed.

**Results:**

Four out of the seven cytologic cases were inconcordance with the histologic diagnosis and cytologically classified as positive for malignant cells, small cell carcinoma, or atypical cells. Three cases showed a characteristic adenoid structure and magenta stroma forming globule, which was distinguished from the four cases. Cytologically, the above mentioned three cases were uniform with relatively small bland nuclei and little cytoplasm. In this study, only one case showed atypical polygonal medium-sized cells with conspicuous nucleoli.

**Conclusions:**

Unlike fine-needle aspiration cytology, magenta stroma globules might offer an alternate clue for cytodiagnosis of AdCC clinically. Bronchial brushings cytology was more present in bland uniform cells with high nuclear to cytoplasmic ratios and background mucoid substance. More cases should be collected and confirmed using histopathology with careful film reading to reduce the rate of misdiagnosis.

## Background

Primary adenoid cystic carcinoma (AdCC) of the tracheobronchial tree is a low malignant tumor, which originates from the submucosal glands of the trachea and bronchus and constitutes only 2% of all respiratory tract tumors [1]. AdCC typically grows slowly and is indolent in its clinical course when compared with that of other primary tracheobronchial neoplasms, but it is a relentlessly recurring and progressive tumor that is easily misdiagnosed [2]. With increasing demand for less invasive procedures and more precise sub-classifications of neoplasms for molecular-targeted therapy, cytology methods are increasingly employed for both diagnosis and prognosis. Unlike transbronchial fine needle aspiration, bronchial brushing is minimally invasive, safe, cost-effective, convenient and particularly useful in diagnosing tracheobronchial neoplasms [3]. In general, bronchial brushing has the highest diagnostic yield of the exfoliative modalities with sensitivities ranging from 35 to 70% and with an overall specificity of 80%, when compared to those in transbronchial biopsy [4]. The purpose of this study was to report seven cases of AdCC of the tracheobronchial tree and review the cytopathological features of bronchial brushing smears and their corresponding histology. We have tried to identify cytopathological features that could potentially help reduce the missed diagnosis rate of AdCC.

## Materials and methods

We searched for seven cases of patients with a diagnosis of primary adenoid cystic carcinoma (AdCC) of the tracheobronchial tree at Zhejiang Provincial People’s Hospital and Jiujiang First People’s Hospital from September 2016 to October 2022. All the cases were linked to surgical excisions in which a previous bronchial brushing was performed that showed abnormal cytology. Clinicopathological and radiological reports for each patient were analyzed to collect pertinent information, including age, gender, smoking history, symptoms, carcinoma sites, metastasis locations, original cytology diagnosis and histology. Cytopathologic specimens were examined by elastic bronchoscopy. The endoscope possessed a channel through which the cytology brush was inserted. Under visual guidance, the brush was swiped over the lesion’s respiratory mucosa and subsequently smeared onto slides. Smears of the gelatinous material were fixed immediately in 95% ethyl alcohol for hematoxylin and eosin (HE) staining. Histopathologic specimens from the bronchoscope or from surgical resections were fixed in 10% buffered formalin, embedded in paraffin, sectioned and stained with HE. The immunohistochemical workup included CD117, P63, Calponin, Ki-67, SMA, S-100, GFAP, CK8/18, TTF-1, NapsinA, CD56 and CgA. All cytology smears and corresponding surgical specimens were carefully studied blinded, by two pathologists (Z.B. and P.L.Z). During this study, we evaluated and recorded the presence of cytomorphological features that could point to a correct preoperative diagnosis in comparison with the histological diagnosis. The analysis focused on cytomorphological features, the presence or absence of magenta stroma globules, background mucoid substance, cell size and arrangement of cellular groups, amount of cytoplasm, cellular border and nuclear features. In addition, dedifferentiated features of large nuclei and conspicuous nucleoli were also assessed and recorded.

## Results

### Clinical information

**Fig. 1:**
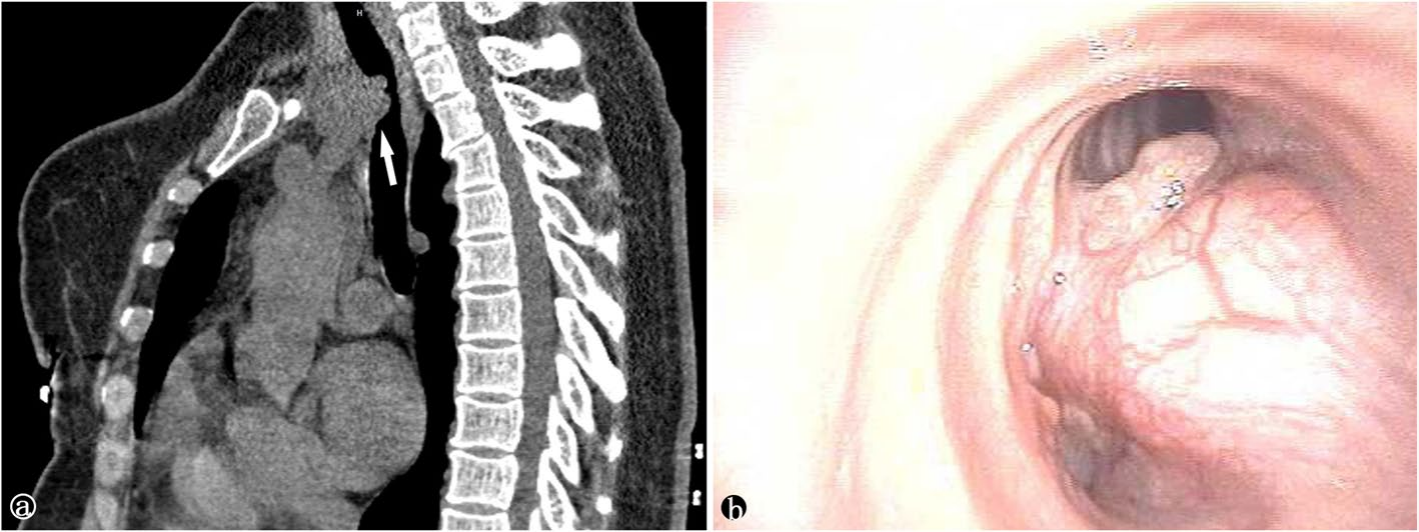
**a** CT scan of neck revealed a mass attached to the tracheal. **b** Adenoid cystic carcinoma almost complete obliteration of the lumen of the bronchus

We identified seven cases of patients with AdCC of the tracheobronchial tree, including two males and five females, age 42–71 years with an average age of 55.7 years. Two patients had a history of smoking, and five patients were symptomatic. The most common symptoms were cough with sputum and exertional dyspnea. Computed tomography imaging and endoscopic evaluation were performed for all patients. In two patients, AdCC appeared in the trachea (Fig. 1a); in one patient, it appeared in the carina, while occurring in the bronchi in four patients (Fig. 1b). Three patients had metastatic lesions in the lymph nodes or lungs. Our original cytologic diagnoses of the seven patients were as follows: AdCC in three cases, positive for malignant cells in two cases, small cell carcinoma in one case, and atypical cells in one case. The relevant clinicopathologic data for all cases analyzed are summarized in Table 1.

**Table 1:** Clinicopathologic features of 7 patients with AdCCs of the tracheobronchial tree

No.	Age /sex	Smoke	Symptom	Site	Metastasis location	Original cytologic diagnosis	Histology (AdCC)
1	42/F	No	Cough with sputum	Trachea	No	AdCC	Cribriform predominant
2	49/F	NA	Cough	Left main bronchus	No	Atypical cells	Cribriform predominant
3	53/M	Yes	Cough/Exertional dyspnea	Trachea	Lung	AdCC	Cribriform predominant
4	54/F	No	Wheezing	Carina	NA	Small cell carcinoma	Solid predominant
5	58/F	No	Cough with sputum/ Haemoptysis	Left main bronchus	Lung/ lymph node	Positive for malignant cells	Solid predominant
6	63/M	No	No	Left main bronchus	No	AdCC	Tubular predominant
7	71/F	Yes	Exertional dyspnea	Right main bronchus	lymph node	Positive for malignant cells	Cribriform predominant

*F* Female, *M* Male, *NA* Not available

### Cytologic features

**Fig. 2:**
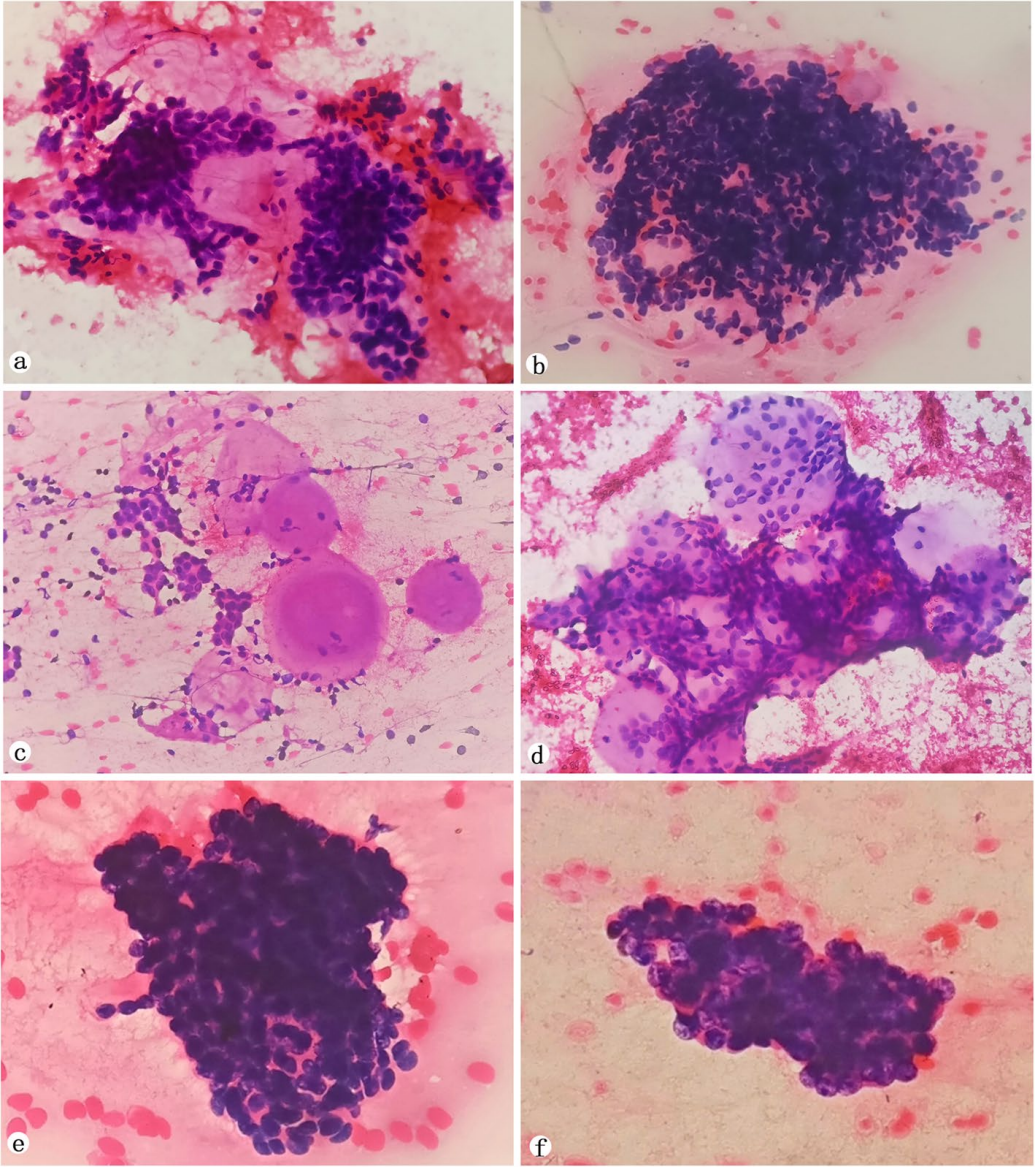
Cytomorphologic features of adenoid cystic carcinoma. **a** Small basaloid-type cells were seen between ciliated columnar epithelial cells, and a mucoid substance was seen in the background (x 200). **b** Irregular clumps of cells with small bland nuclei and inconspicuous nucleoli. b. The mucoid substance was almost indiscernible in the background (x 200). **c** The magenta stroma globules is easily detected and is deprived of cells (x 200). **d** Tubular structures constituted by basaloid cells surrounding a magenta stroma globule (x 200). **(e)** Atypical and crowding cells with scant cytoplasm and inconspicuous nucleoli (x 400). **f** A clusters of medium-sized cells with coarse chromatin, and conspicuous nucleoli (x 400)

The cellular smears showed fragments of small basaloid-type cells between the ciliated columnar epithelial cells and a mucoid substance in the background (Fig. 2a). The relatively large number of carcinomatous cells were uniform with a small size, a round and ellipsoidal shape, aggregating to form irregular clumps of cells with a possibility of scattering. The cell’s cytoplasm was extremely scanty and basophilic. There were dense, relatively small and bland nuclei with chromatin and there were inconspicuous nucleoli (Fig. 2b). Massive, round and well-demarcated acellular globules of magenta stroma were found in three cases (Fig. 2c). Magenta stroma globules surrounded by the previously described basaloid cells were also observed (Fig. 2d). In one of the cases, carcinomatous cells were crowded with numerous naked nuclei and the chromatin was dense, which was considered to be small cell carcinoma in the original cytologic diagnosis (Fig. 2e). A few cohesive clusters of repetitive medium-sized cells, with coarse chromatin and conspicuous nucleoli were also found (Fig. 2f). However, this did not meet the diagnostic criteria for dedifferentiated carcinoma.

### Histologic findings

**Fig. 3:**
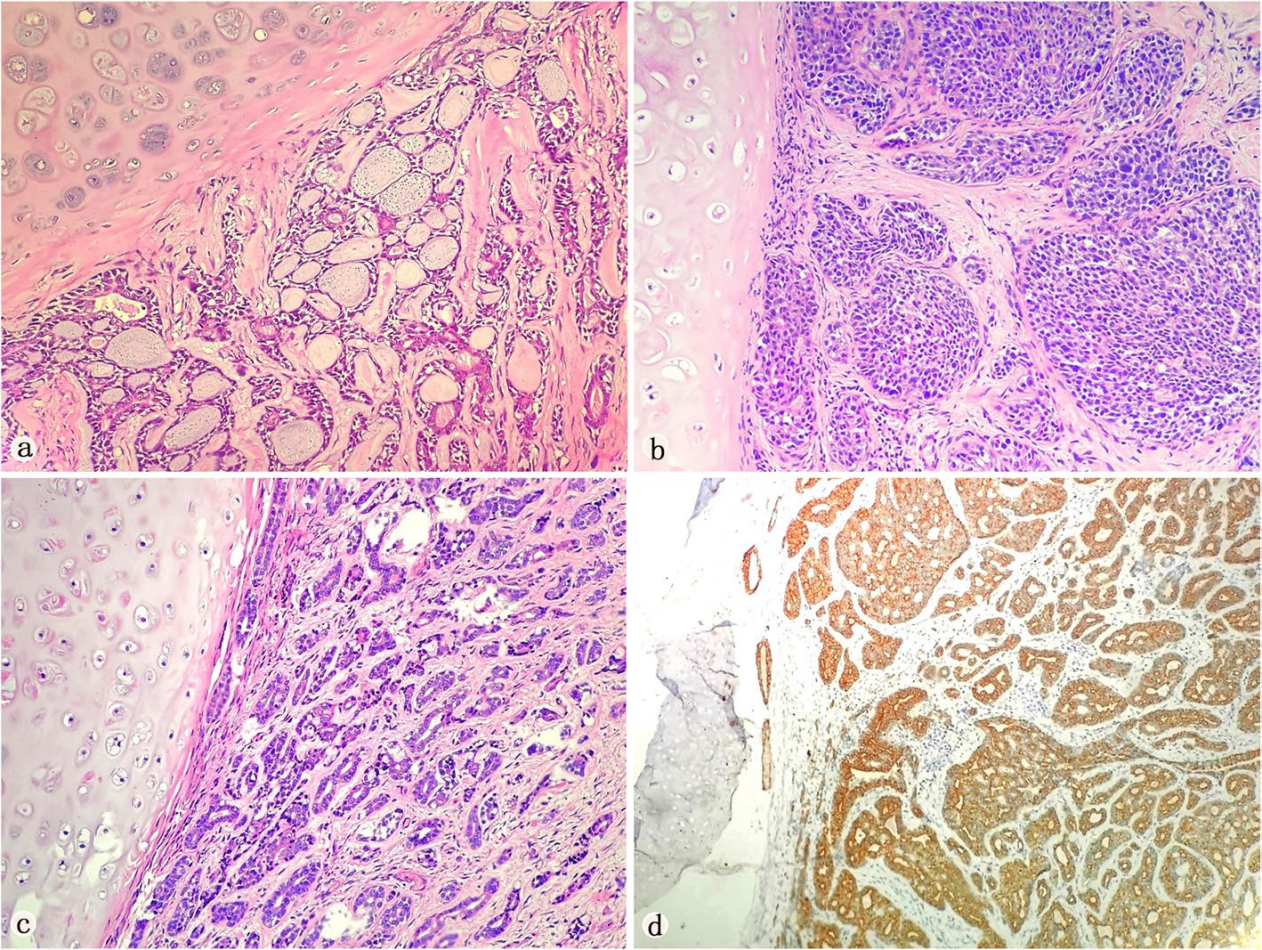
Histomorphologic features of adenoid cystic carcinoma. **a** A cribriform pattern showing acellular spaces containing mucoid and hyaline material (x 200). **b** A solid pattern demonstrated diffuse tumor cells with heavy nuclear chromatin distribution(x 200). **c** A tubular pattern with excessive extracellular basal lamina material and mucinous material (x 200). **d** CD117 showing positive in epithelial cells (x 200)

Surgical specimens by lobectomy procedures and by biopsy were collected in six cases and one case, respectively. Tumor size varied from 1.5 cm to 4.5 cm, with a mean of 3.2 cm. Lymph node and lung metastases were present in three patients. Microscopy showed that the tumor consisted of a double-layer structure with basaloid epithelial and myoepithelial cells. The tumor cells were small and uniform in size, had little cytoplasm, were round or polygonal in shape, and had deep-stained nuclei. However, splintering nuclei were rare and slightly basophilic mucoid material in adenoid cavities and sieve pores was observed. The cells were distributed as cribriform or tubular while a solid pattern tended to predominate in each case: cribriform (*n* = 4; Fig. 3a), solid (*n* = 2; Fig. 3b), and tubular (*n* = 1; Fig. 3c). All tissue samples underwent immunohistochemistry, including tests for CD117 (7, 100%); (Fig. 3d), CK8/18 (7, 100%), P63 (7, 100%), Calponin (6, 85.7%), SMA (6, 85.7%), S-100 (5, 71.4%), GFAP (2, 28.6%) and Ki-67 (index range 2–40%). The remainder of the antibodies of tumor cells was negative. The relevant IHC markers for each antibody for all cases are summarized in Table 2.

**Table 2:** Summary of the results for each antibody for all 7 cases

Antibody	Case 1	Case 2	Case 3	Case 4	Case 5	Case 6	Case 7
CD117	+	+	+	+	+	+	+
CK8/18	+	+	+	+	+	+	+
P63	+	+	+	+	+	+	+
Calponin	+	+	+	-	+	+	+
SMA	+	+	+	-	Focal +	+	+
S-100	+	+	-	+	-	+	+
GFAP	-	Focal +	-	-	-	-	Focal +
TTF-1	-	-	-	-	-	-	-
NapsinA	-	-	-	-	-	-	-
CgA	-	-	-	-	-	-	-
CD56	-	-	-	-	-	-	-
Ki-67	10%	2%	10%	20%	40%	5%	10%

*SMA* Smooth muscle actin, *GFAP* Glial fibrillary acidic protein, *CgA* Human chromogranin A

## Discussion

The cytological diagnosis of adenoid cystic carcinoma has been widely used in fine-needle aspiration of salivary gland tumors. Since the puncture is an invasive examination, the tumor cells obtained are abundant, with little interference. Classic basophilic basaloid epithelial cells and many magenta stroma-forming rounded structures provide convenience for cytological diagnosis of AdCC [5]. The bronchial brush cytology examination can directly brush the tumor cells from the lesion, has the characteristics of a simple operation, little trauma and good repeatability. It is of great significance to the detection, location, diagnosis and follow-up of bronchial primary tumors [6]. However, due to the atypical clinical symptoms, low incidence, and the presence of non-invasive lesions in these examined cases, the internal information of deep tumors cannot be effectively obtained, which is why the bronchoscope brushing cytological diagnosis of AdCC is still difficult and easy to be missed or mistaken [7].

In our study, AdCC is an affliction of middle or old age and female incidence was higher than male. Among the patients, two were smokers. However, the relationship between smoking and AdCC is not clear. At the sites of the disease, the lesions were distributed in the trachea, left and right main bronchi and the bronchial spine. In three of the cases (42.9%), more lesions appeared in the left main bronchus, which was different from that in AdCC cases reported in previous studies [8]. The clinical symptoms presented in AdCC are atypical. Only common respiratory symptoms are present such as cough, asthma, and dyspnea thereby making AdCC a difficulty attention. Therefore, the overall clinical course is longer. Previous studies indicated that the cancer cells didn’t metastasize easily, but in three of the seven cases, lymph node or intrapulmonary metastasis occurred. Most of the findings were discovered late, and the prognosis was poor [9]. In six patients (85.7%), positive cytology in bronchoscopy was observed; three patients (42.9%) were diagnosed as suffering from AdCC. This evidence supports that it is difficult to directly diagnose AdCC by bronchial brush cytology.

Cytological smears of classical bronchial tree AdCC showed uniform basal-like cells, plasma-like cells, scarce cytoplasm, smooth nuclear membrane, high nuclear-to-mass ratio, fine nuclear chromatin and invisible nucleoli [10]. Tumor cells are usually surrounded by round, well-divided transparent or red-stained mucus globular basement membrane materials, showing pseudoadenoid or cluster structure, which is of great significance for a correct cytological diagnosis [11]. In this study, only three of the seven specimens had basal membrane-like globular substances, one of which was especially rich, the other two specimens were less globular, acellular, and an accurate diagnosis could be arrived at after careful observation. Some research reports found that the possibility of diffuse lamellar or transparent globules in bronchial AdCC is relatively small when compared to those in other organs affected by AdCC [5]. Similar results were found in this paper. This may be due to tracheal brushing to remove the surface of the tumor and the inability to penetrate the tumor as opposed to being able to with puncture cytology.

Therefore, when there is no characteristic basement membrane and only mild heteromorphic basal-like cells with a high nucleoplasm ratio, homogeneous chromatin, smooth nuclear membrane but no nucleolus, cytological diagnosis of AdCC is difficult. The remaining four patients in this study were not correctly diagnosed. In one of the cases, cells were round and oval, a little larger than the typical basilar cells. The nuclear chromatin was slightly thicker, there was a small nucleolus, unusual mitosis and a histological examination confirmed solid AdCC. To our knowledge, solid AdCC tracheal cytology shows twice the cell-size of conventional AdCC. Solid AdCCs show morphologically uniform tumor cells, are rich in eosinophilic cytoplasm, and have visible nucleoli with three-dimensional clusters. There is no transparent globular or basal material in the background. This study showed cases with similar characteristics. The prognosis of solid type was poor when compared to that in other types of tumors [12].

In one case, the tumor cells were abundant and crowded and misdiagnosed as small cell carcinoma, and herein lies the difficulty of AdCC bronchial tree cytology. There are similarities in cytology between different types of tumors, but treatment and prognosis are very different. To our knowledge, small cell carcinomas have greater heterogeneity of the cells relative to AdCC, and nuclear molding and ‘crush artifact’ are typical findings. Mitotic figures are found easily. Due to the invariable necrosis, a background of granular debris is to be expected [13]. Furthermore, it is believed that small cell carcinoma is closely related to smoking history, and the clinical symptoms of paraneoplastic syndromes are not uncommon [14]. In a retrospective review of this study, one case was missed: a small number of scattered basal-like tumor cells were thought to be metaplastic or reactive ciliated columnar epithelial cells. Some researchers believe that reactive cells generally do not form clusters, and have a small volume, the presence of cilia and chromatin is relatively light. Combined with inflammatory background and clinical imaging, benignity is considered. A case of tracheal dedifferentiation AdCC cytology reported by Japanese scholars, Cells are pleomorphic, have large nuclei (more than three times the size of conventional low-grade AdCC), large chromatin and the distinct features of nucleoli [15]. The above features were not found in this collection.

The differential diagnosis of AdCC includes several tumors originating from tracheobronchial tree [1, 16]. Cribriform and tubular AdCC is sometimes difficult to distinguish it from epitheial-myoepthelial carcinoma, mucoepidermoid carcinoma and adenocarcinoma. Epithelial-myoepithelial carcinoma has a well defined biphasic appearance. The myoepithelial cells often have a cleared cytoplasm, while CD117 is negative. Mucoepidermoid carcinoma with larger mucous and intermediate cells, but p63, SMA and Calponin are usually absent. Adenocarcinoma is more heterogeneous and neoplastic cells are shown with abundant vacuolated cytoplasm, while is TTF‑1 and Napsin A strongly positive. Solid AdCC without identifiable adenoid structure and extracellular stroma could potentially be misinterpreted as basaloid squamous cell carcinoma and neuroendocrine carcinoma, especially in a limited sample. Basaloid squamous cell carcinoma is more heterogeneous, with squamous cell differentiation. Additionally, the immunohistochemical profile shows little to absent CK8/18,S-100 and SMA reactivity. Neuroendocrine carcinoma showing a high nuclear to cytoplasmic ratio, salt-and-pepper nuclear chromatin distribution, high mitotic rate and tumor necrosis. Immunohistochemistry can be helpful, as AdCC are negative for syn, CgA and CD56 reactivity. Finally, sufficient sampling can be helpful in diagnostically difficult cases.

In summary, there are some difficulties in the diagnosis of AdCC by bronchoscopic cytology, especially when the tumor does not infiltrate through the mucosa or loses the characteristic basement membrane-like substance. The smear should be observed more carefully, according to the microscopic characteristics of basal-like cells. Additionally, hospitals can improve the AdCC diagnosis rate using cytology by using transbronchial fine needle aspiration. It is best to combine biopsies with immunohistochemical stains observed in order to make a comprehensive judgment.

## Data Availability

Supporting data can be requested by emailing the corresponding author for reasonable claims.
